# Prediction of Hemorrhagic Transformation After Ischemic Stroke: Development and Validation Study of a Novel Multi-biomarker Model

**DOI:** 10.3389/fnagi.2021.667934

**Published:** 2021-05-28

**Authors:** Junfeng Liu, Yanan Wang, Yuxi Jin, Wen Guo, Quhong Song, Chenchen Wei, Jing Li, Shanshan Zhang, Ming Liu

**Affiliations:** ^1^Department of Neurology, Center of Cerebrovascular Diseases, West China Hospital, Sichuan University, Chengdu, China; ^2^West China School of Medicine, Sichuan University, Chengdu, China; ^3^Department of Neurology, The Affiliated Hospital of Qingdao University, Qingdao, China; ^4^Department of Neurology, The First People’s Hospital of Ziyang, Ziyang, China; ^5^Department of Neurology, Mianyang Central Hospital, Mianyang, China

**Keywords:** acute ischemic stroke, multiple biomarkers, hemorrhagic transformation, risk reclassification, prediction model

## Abstract

**Objectives**: We aimed to develop and validate a novel multi-biomarker model for predicting hemorrhagic transformation (HT) risk after acute ischemic stroke (AIS).

**Methods**: We prospectively included patients with AIS admitted within 24 h of stroke from January 1st 2016 to January 31st 2019. A panel of 17 circulating biomarkers was measured and analyzed in this cohort. We assessed the ability of individual circulating biomarkers and the combination of multiple biomarkers to predict any HT, symptomatic HT (sHT) and parenchymal hematoma (PH) after AIS. The strategy of multiple biomarkers in combination was then externally validated in an independent cohort of 288 Chinese patients.

**Results**: A total of 1207 patients with AIS (727 males; mean age, 67.2 ± 13.9 years) were included as a derivation cohort, of whom 179 patients (14.8%) developed HT. The final multi-biomarker model included three biomarkers [platelets, neutrophil-to-lymphocyte ratios (NLR), and high-density lipoprotein (HDL)] from different pathways, showing a good performance for predicting HT in both the derivation cohort (c statistic = 0·64, 95% CI 0·60–0·69), and validation cohort (c statistic = 0·70, 95% CI 0·58–0·82). Adding these three biomarkers simultaneously to the basic model with conventional risk factors improved the ability of HT reclassification [net reclassification improvement (NRI) 65.6%, *P* < 0.001], PH (NRI 64.7%, *P* < 0.001), and sHT (NRI 71.3%, *P* < 0.001).

**Conclusion**: This easily applied multi-biomarker model had a good performance for predicting HT in both the derivation and external validation cohorts. Incorporation of biomarkers into clinical decision making may help to identify patients at high risk of HT after AIS and warrants further consideration.

## Introduction

Hemorrhagic transformation (HT) is not only a part of the natural history of ischemic stroke, but also a major complication of reperfusion therapy, and is associated with increased morbidity and mortality (Álvarez-Sabín et al., 2013). A recent meta-analysis (Sadeh-Gonik et al., 2018) reported a HT and symptomatic HT (sHT) rates of 24% and 8%, respectively, when patients treated with thrombectomy. In a survey of emergency physicians about thrombolysis, Brown et al. (2005) reported about two thirds of physicians may overestimate the risk of HT, and so they inappropriately avoid thrombolysis. In high-income countries, only 20% of ischemic stroke patients received thrombolysis (Langhorne et al., 2018), and less than 3% of patients in China (Wu et al., 2019). In addition, our previous review (Wu et al., 2019) suggested that a concern over HT risk also hampers the widespread adoption of anticoagulation for secondary prevention in patients with atrial fibrillation (AF) in Asia, especially in China. Therefore, early and quickly identification of patients at high risk of HT to help individualized treatment decisions in patients with ischemic stroke is a top priority of research on stroke.

At present, evaluating the risk of HT after ischemic stroke mainly depends on clinical factors and neuroimaging (Álvarez-Sabín et al., 2013). By combining those clinical and neuroimaging markers, several risk models (Cucchiara et al., 2008; Lou et al., 2008; Menon et al., 2012; Strbian et al., 2012; Saposnik et al., 2013) have been developed to stratify the risk of HT after thrombolysis. However, existing models have only a modest capability in predicting those at “high risk” for HT after stroke (Sung et al., 2013), irrespective of the use of thrombolysis. In addition, some neuroimaging markers used in these models may not be routinely available in all ischemic stroke patients due to contraindications and lack of availability. Hence, there has been increasing interest to find new approaches which can achieve better accuracy and be easier to use in predicting HT. Circulating biomarkers may be a promising approach.

A growing number of studies reported that individual novel biomarkers, such as matrix metalloproteinase 9, S100B, and tight-junction proteins, were associated with HT after ischemic stroke (Montaner et al., 2003; Foerch et al., 2007; Kelly et al., 2008; Kazmierski et al., 2012; Yuan et al., 2018). However, there are multiple practical challenges before these novel biomarkers can be used in the clinical practice, including the need for high sensitivity and specificity, and fast and easy-to-assess availability of the results. Furthermore, few studies investigated the relationship between multiple biomarkers and HT systematically, and the clinical significance of multiple biomarkers when used in combination for predicting HT is also unknown. Therefore, we aimed to develop and externally validate a novel multi-biomarker model for predicting HT after ischemic stroke that can be calculated from readily available routine clinical circulation biomarkers.

## Materials and Methods

### Participants

A prospective cohort of 1,362 patients with ischemic stroke (≥18 years old) admitted to West China hospital, Sichuan University within 24 h after stroke onset between January 1st 2016 and January 31st 2019, were recruited as a derivation cohort. Patients with hemorrhage on the initial admission imaging (*n* = 33), those who did not have a follow-up computed tomography (CT) or magnetic resonance imaging (MRI) scan within 7 days after admission (*n* = 118) and those without data of all 17 circulating biomarkers (*n* = 4) were excluded, leaving 1,207 patients for the final analysis.

We also screened 350 patients with ischemic stroke admitted to Mianyang Central Hospital and The First People’s Hospital of Ziyang within 24 h after stroke onset between June 1st 2017 and March 31st 2019 as an external validation cohort. The cohort excluded patients with hemorrhage on the initial admission imaging (*n* = 2), those who did not have a follow-up CT or MRI scan during hospitalization (*n* = 53) and those without data of all 17 circulating biomarkers (*n* = 7). Two-hundred and eighty-eight patients were included for the final analysis of external validation.

The definition of ischemic stroke was based on the World Health Organization criteria (Aho et al., 1980), and further confirmed by a CT or MRI scan (Sacco et al., 2013). The study was approved by the Biomedical Research Ethics Committee of West China Hospital, Sichuan University [2016(339)]. Informed consent was obtained from all patients or their relatives.

### Data Collection

Baseline data on demographic characteristics (age and sex), vascular risk factors (hypertension, diabetes mellitus, atrial fibrillation, hyperlipidemia, smoking and drinking), NIHSS score on admission, therapy before and after admission, and the Trial of ORG 10172 in Acute Stroke Treatment (TOAST) classification were collected using a standard questionnaire. Systolic blood pressure (SBP) and diastolic blood pressure (DBP) were measured and obtained at admission.

### Circulating Biomarker Measurements

The circulating biomarkers were selected based on the results of our (Wang et al., 2020a, b) and previous review (Lu et al., 2018). Accordingly, 17 biomarkers representing inflammation [white blood cells, WBC, monocyte, neutrophils, lymphocytes, and neutrophil-to-lymphocyte ratios (NLR)], coagulation/fibrinolysis disorder (platelets), vasoreactivity (creatinine, and cystatin C), lipid metabolism [total cholesterol (TC), triglyceride (TG), high-density lipoprotein (HDL), low-density lipoprotein (LDL)], liver function [alkaline phosphatase (ALP), alanine aminotransferase (ALT), aspartate aminotransferase (AST) and total bilirubin (TBIL)] and glucose were measured and analyzed in patients with acute ischemic stroke (AIS).

The median time from stroke onset to biomarker blood draw was 4.0 h (2.4, 6.8 h interquartile range). All circulating biomarkers were measured in the department of laboratory medicine by experienced technicians who were blinded to the clinical characteristics of the patients. The NLR was calculated as the ratio of neutrophil absolute value to lymphocyte absolute value, both obtained from the same blood sample.

### Definition of Hemorrhagic Transformation

Any hemorrhage found on the follow-up CT or MRI within 7 days after admission but not detected on initial head CT was defined as HT. sHT was diagnosed according to the definition of the National Institute of Neurological Disease and Stroke study (National Institute of Neurological Disorders and Stroke rt-PA Stroke Study Group, 1995). Two trained neurologists who were blinded to patients’ clinical data assessed HT and sHT independently, and a third neurologist was consulted in case of disagreement. The inter-rater reliability (Kappa) for assessing the presence of HT and sHT was 0.92 and 0.87, separately. HT was also classified as Hemorrhagic Infarction (HI) and Parenchymal Hematoma (PH) according to the European Cooperative Acute Stroke Study III definitions (de Los Ríos la Rosa et al., 2012).

### Outcomes

The primary outcome of this analysis is the composite of sHT and asymptomatic HT detected during hospitalization (i.e., any HT). We chose a composite outcome because, although sHT was the most feared complication of reperfusion therapy in AIS (Yaghi et al., 2017), asymptomatic HT was found to worsen long-term clinical outcomes (Lei et al., 2014), and also the main concern of initiating anticoagulants at discharge for secondary prevention in ischemic stroke patients with atrial fibrillation in China (Liu et al., 2021). Moreover, an increased number of primary outcomes was desirable to support the statistical power of the analysis. The secondary outcomes of the study were the presence of PH and sHT.

### Model Development and External Validation

In the derivation cohort, univariate logistical analysis was carried out to investigate the relationships between biomarkers and HT. Variables that showed a univariate relationship with HT (*P* < 0.05) were further analyzed in multivariate logistic regression analysis. Each biomarker was assessed separately using logistic analysis.

Multiple biomarkers were evaluated using both a categorical and continuous exposure measure. Only biomarkers significantly associated with HT in the initial stage were included in the following analysis. First, receiver operating characteristics (ROC) curve analysis was used to identify the optimal cut-off point of each biomarker, and the effect of multiple biomarkers in combination on HT was evaluated using a multivariable analysis. Second, we created a continuous multi-marker score based on an equation reported by a previous study (Zhong et al., 2019): H = (β1 × biomarker A) + (β2 × biomarker B) + (β3 × biomarker C), and so on. β1, β2, and β3 represented the estimates of beta coefficients for biomarkers A, B, and C, and were obtained by the logistic regression model for the study outcome. Patients were divided based on the tertiles of the multi-biomarker score. Odds radio (ORs) and 95% confidence intervals (CIs) for each group were calculated and reported using the lowest tertile as reference. Tests for linear trends in ORs were conducted using the number of biomarkers that achieved the cut-off points or the median within each tertile as the predictor. We further used a logistic regression model with restricted cubic splines to evaluate the pattern and magnitude of the association between multi-biomarker score and the risk of HT with three knots (at the 10th, 50th, and 90th percentiles). The discriminatory ability of one or multiple biomarkers in combination for predicting HT were also evaluated in the external validation cohort.

### The Predictive Value of Adding Circulating Biomarkers to the Conventional Model

A conventional model was built based on the multivariate logistic regression analysis for the association of traditional risk factors (such as age and NIHSS score) with HT. The ability to reclassify HT risks by adding each biomarker and the combination of multiple biomarkers/the multi-biomarker score to the conventional model was also evaluated. We calculated the C statistics, net reclassification improvement (NRI) and integrated discrimination improvement (IDI) to assess the risk refinement (enhanced risk differentiation) provided by one or more new markers (Pencina et al., 2008). The C statistics of each model were compared by Delong method (DeLong et al., 1988). Likelihood ratio tests were then performed to evaluate whether the global model fit improved after the addition of biomarkers.

### Statistical Analysis

Continuous data were reported as mean ± standard deviation or median with interquartile range, while categorical data were reported as frequencies and percentages. Differences in categorical data were assessed using *χ*^2^ test, while differences in continuous data were assessed using student’s *t*-test or the Mann–Whitney *U* test, as appropriate. A 2-sided *P* < 0.05 was considered statistically significant. Statistical analysis was performed using SPSS 25.0 (IBM, Chicago, IL, USA) and STATA 15.0 (Corporation, College Station, TX, USA).

### Data Availability

The data that support the results of this study are available from the corresponding author upon reasonable request.

## Results

**Figure 1:**
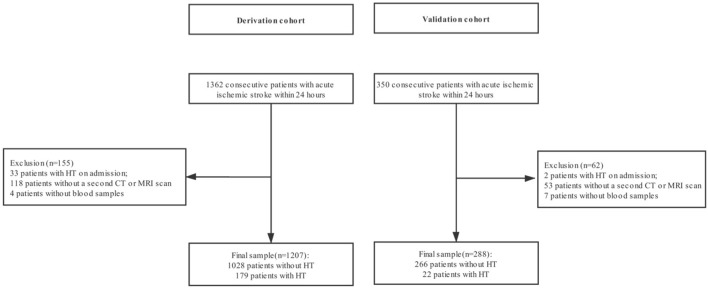
Flowchart of patients inclusion. HT, hemorrhagic transformation; CT, computed tomography; MRI, magnetic resonance imaging.

One-thousand, two-hundred and seven patients with AIS (60.2% males; mean age, 67.2 ± 13.9 years) were included as a derivation cohort, and 288 patients (62.9% males; mean age, 69.0 ± 12.0 years) as an external validation cohort (Figure 1). The demographic and clinical characteristics are summarized in Supplementary Table 1. During hospitalization, 179 (14.8%) patients were found to have HT, of whom 76 (6.3%) with HI, 103 (8.5%) with PH, and 44 (3.7%) with sHT were in the derivation cohort. The median time of stroke onset to develop HT was 3 days (1–4 days), and 95 patients (53.1%) were diagnosed with HT on CT scans. In the external cohort, 22 patients (7.6%) had HT. Nighteen (6.6%) with HI, 3 (1.0%) with PH, and 6 (2.1%) with sHT were detected. The median time of stroke onset to develop HT was 2 days (1–4 days), and nine patients (40.9%) were found to have HT on CT scans.

### Single Biomarker and Association With Hemorrhagic Transformation

Univariable analysis in the derivation cohort (Table 1) found a significant association of HT with several conventional predictors that were consistently reported previously: age, sex, atrial fibrillation, National Institutes of Health Stroke Scale score, smoking, drinking, SBP, antiplatelets, lipid-lowering agents, thrombolysis/thrombectomy treatment and Toast classification. Nine biomarkers were also identified to associate with HT significantly in the univariable analysis. After adjusting for confounders, the levels of platelets, neutrophils, lymphocytes, NLR, and HDL were significantly and independently related to risk of HT (all *P* < 0.05, Table 2).

**Table 1:** Univariable analysis in the derivation cohort.

	No HT (*n* = 1,028)	HT (*n* = 179)	*P*-values
**Demographics**			
Age (years), mean (SD)	66.68 (14.07)	70.34 (12.77)	0.001^c^
Male, *n* (%)	642 (62.45%)	85 (47.49%)	<0.001^b^
**Medical history**			
Hypertension, *n* (%)	562 (54.67%)	94 (52.51%)	0.593^b^
Diabetes mellitus, *n* (%)	208 (20.23%)	38 (21.23%)	0.76^b^
Hyperlipidemia, *n* (%)	34 (3.31%)	4 (2.23%)	0.448^b^
quad Atrial fibrillation, *n* (%)	151 (14.69%)	56 (31.28%)	<0.001^b^
**Therapy before admission**
Antiplatelets, *n* (%)	122 (11.87%)	16 (8.94%)	0.256^b^
Lipid-lowering agents, *n* (%)	70 (6.81%)	11 (6.15%)	0.743^b^
Anticoagulants, *n* (%)	60 (5.84%)	11 (6.15%)	0.871^b^
**Clinical features**
Onset to addition time (hours), median (IQR)	6.00 (3.00–24.00)	5.00 (3.00–15.00)	0.002^a^
Current smoking, *n* (%)	426 (41.44%)	59 (32.96%)	0.033^b^
Current drinking, n (%)	256 (24.90%)	30 (16.76%)	0.018^b^
NIHSS on admission, median (IQR)	5.00 (2.00–12.00)	14.00 (9.00–20.00)	<0.001^a^
SBP (mmHg), mean (SD)	148.43 (23.97)	142.93 (22.92)	0.004^c^
DBP (mmHg), mean (SD)	85.16 (15.65)	84.32 (17.13)	0.513^c^
**Biomarkers**			
Glucose (mmol/L), mean (SD)	7.99 (3.35)	8.23 (2.53)	0.362^c^
WBC count (× 10^9^/L), median (IQR)	10.10 (7.70–13.70)	11.60 (8.68–15.20)	0.002^a^
Monocyte count (× 10^9^/L), median (IQR)	0.36 (0.27–0.47)	0.34 (0.25–0.48)	0.082^a^
Neutrophils (× 10^9^/L), median (IQR)	5.36 (4.03–7.43)	6.51 (4.76–8.32)	<0.001^a^
Lymphocytes, median (IQR)	1.33 (0.98–1.82)	1.11 (0.82–1.63)	<0.001^a^
Neutrophil-to-lymphocyte Ratio, median (IQR)	3.97 (2.48–6.87)	5.71 (3.40–9.40)	<0.001^a^
TG (mmol/L), median (IQR)	1.32 (0.92–1.93)	1.13 (0.78–1.46)	<0.001^a^
TC (mmol/L), median (IQR)	4.32 (3.65–5.04)	4.23 (3.46–4.77)	0.068^a^
HDL (mmol/L), median (IQR)	1.22 (0.99–1.47)	1.32 (1.07–1.55)	0.003^a^
LDL (mmol/L), median (IQR)	2.58 (1.98–3.23)	2.46 (1.90–2.98)	0.019^a^
Platelets (×10^9^/L), median (IQR)	169.50 (135.00–211.25)	151.00 (107.50–188.00)	<0.001^a^
Bilirubin (10^−6^ mol/l), median (IQR)	7.85 (6.26–10.43)	8.43 (6.75–10.41)	0.068^a^
Alanine aminotransferase (IU/L), median (IQR)	19.00 (13.00–27.25)	19.00 (14.00–26.00)	0.736^a^
Aspartate aminotransferase (IU/L), median (IQR)	22.00 (18.00–27.00)	22.00 (18.00–28.50)	0.234^a^
Alkaline phosphatase (IU/L), median (IQR)	78.00 (65.00–94.00)	79.00 (64.50–94.50)	0.839^a^
Serum creatinine, median (IQR)	74.00 (62.00–89.00)	71.00 (57.00–85.00)	0.015^a^
Cystatin C (mg/L), median (IQR)	0.93 (0.80–1.13)	0.91 (0.78–1.15)	0.288^a^
**Therapy after admission**			
Antiplatelets, *n* (%)	941 (91.54%)	146 (81.56%)	<0.001^b^
Anticoagulants, *n* (%)	151 (14.69%)	26 (14.53%)	0.954^b^
Lipid-lowering agents, *n* (%)	945 (91.93%)	149 (83.24%)	<0.001^b^
Thrombolysis, *n* (%)	102 (9.92%)	28 (15.64%)	0.023^b^
Thrombectomy, *n* (%)	85 (8.27%)	36 (20.11%)	<0.001^b^
EVT, *n* (%)	165 (16.05%)	56 (31.28%)	<0.001^b^
**TOAST classification**			
Large-artery atherosclerosis, *n* (%)	303 (29.47%)	51 (28.49%)	<0.001^b^
Small-artery occlusion, *n* (%)	259 (25.19%)	1 (0.56%)	
Cardioembolic, *n* (%)	254 (24.71%)	95 (53.07%)	
Undetermined etiology, *n* (%)	185 (18.00%)	28 (15.64%)	
Other etiology, *n* (%)	27 (2.63%)	4 (2.23%)	

*Abbreviations: HT, hemorrhagic transformation; NIHSS, National Institutes of Health Stroke Scale; SBP, systolic blood pressure; DBP, diastolic blood pressure; WBC, white blood cell; TG, triglyceride; TC, total cholesterol; HDL, high-density lipoprotein cholesterol; LDL, low-density lipoprotein cholesterol; EVT, endovascular treatment (Thrombolysis/Thrombectomy); TOAST, the Trial of ORG 10172 in Acute Stroke Treatment. ^a^Mann–Whitney Test. ^b^*χ*^2^ test. ^c^Student’s test*.

**Table 2:** Biomarkers and risk of hemorrhagic transformation after acute ischemic stroke.

Biomarkers	Adjusted^a^	
	OR (95% CI)	*P*-value
Platelets (× 10^9^/L)	0.99 (0.99, 1.00)	<0.001
HDL (mmol/L)	1.95 (1.23, 3.07)	<0.001
Neutrophil-to-lymphocyte Ratio	1.04 (1.01, 1.07)	<0.001
Neutrophils (× 10^9^/L)	1.06 (1.00, 1.12)	0.04
Lymphocytes	0.69 (0.53, 0.89)	<0.001
TG (mmol/L)	0.88 (0.73, 1.05)	0.15
LDL (mmol/L)	0.98 (0.80, 1.20)	0.87
WBC count (× 10^9^/L)	1.03 (0.97, 1.09)	0.30
Serum creatinine (umol/L)	1.00 (1.00, 1.00)	0.51

*Abbreviations: OR, odds radio; CI, confidence level; HDL, high-density lipoprotein cholesterol; TG, triglyceride; LDL, low-density lipoprotein cholesterol; WBC, white blood cell^a^ adjusted for age, sex, atrial fibrillation, National Institutes of Health Stroke Scale score, smoking, drinking, systolic blood pressure, antiplatelets, lipid-lowering agents, endovascular treatment (Thrombolysis/Thrombectomy) and the Trial of ORG 10172 in Acute Stroke Treatment*.

### Multi-biomarkers and Association With Hemorrhagic Transformation

**Figure 2:**
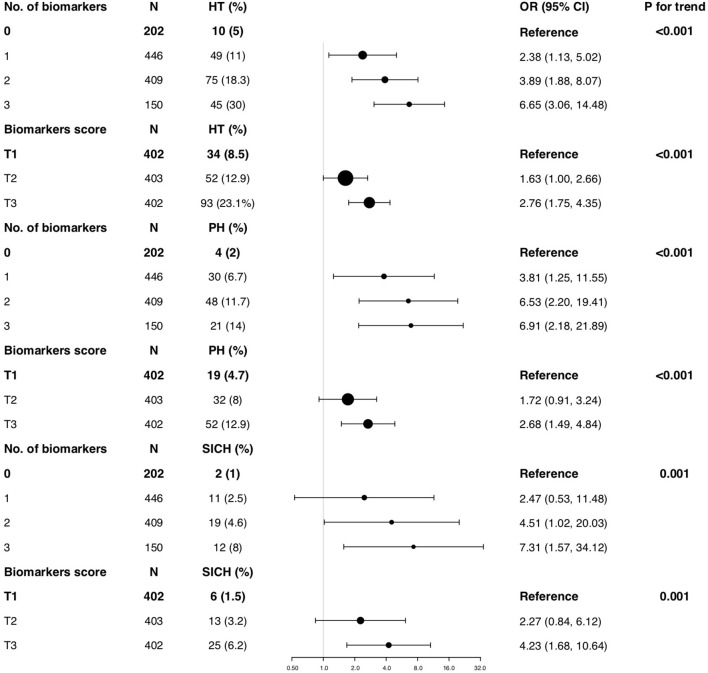
Multivariable-adjusted odds radios (ORs; 95% CIs) of HT according to the number of elevated biomarkers or tertiles of biomarkers scores among acute ischemic stroke (AIS) patients. Odds ratios for HT were adjusted for age, sex, atrial fibrillation, NIHSS, smoking, drinking, Systolic blood pressure (SBP), antiplatelets, lipid-lowering agents, Thrombolysis/Thrombectomy treatment and TOAST classification. Odds ratios for PH were adjusted for age, sex, atrial fibrillation, NIHSS, smoking, drinking, SBP, antiplatelets, lipid-lowering agents, Thrombolysis/Thrombectomy treatment and TOAST classification. Odds ratios for sICH were adjusted for atrial fibrillation, NIHSS and Thrombolysis/Thrombectomy treatment. HT, hemorrhagic transformation; PH, Parenchymal hematoma; sICH, Symptomatic intracerebral hemorrhage; NIHSS, National Institutes of Health Stroke Scale score; TOAST, the Trial of ORG 10172 in Acute Stroke Treatment.

ROC curve analysis was used to identify the optimal cut-off points of platelets, neutrophils, lymphocytes, NLR, and HDL for predicting HT (Supplementary Table 2). To avoid collinearity in the multiple biomarkers model, only one biomarker from the same pathway underlying HT was chose based on the ROC curve for the following analysis. Accordingly, platelets, NLR, and HDL were included in the following multi-biomarker analysis. The prevalence of HT according to the number of biomarkers achieving the thresholds or tertiles of the multi-biomarker score are shown in Figure 2.

**Figure 3:**
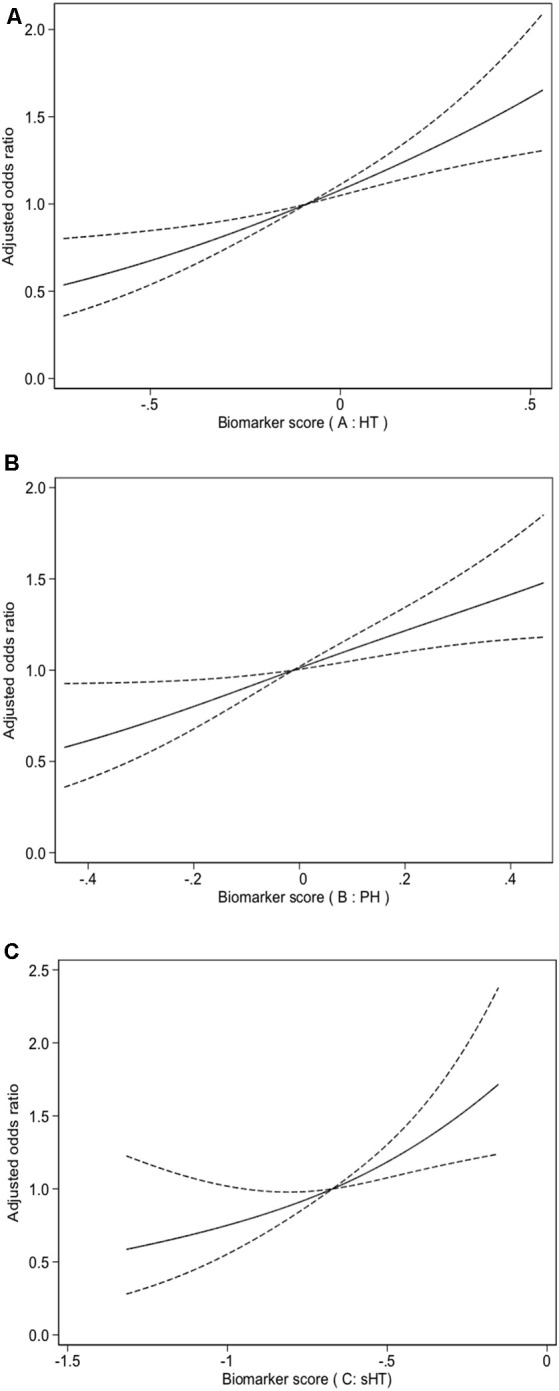
Multiple spline regression analyses were used to analyze the association between biomarkers score and HT **(A)**, PH **(B)**, sHT **(C)** with three knots (at the 10th, 50th, 90th percentiles). Solid line indicates adjusted odds ratios, and dotted line indicates 95% confidence intervals (CIs). Odds ratios for HT were adjusted for age, sex, atrial fibrillation, NIHSS, smoking, drinking, SBP, antiplatelets, lipid-lowering agents, Thrombolysis/Thrombectomy treatment and TOAST classification. Odds ratios for PH were adjusted for age, sex, atrial fibrillation, NIHSS, smoking, drinking, SBP, antiplatelets, lipid-lowering agents, Thrombolysis/Thrombectomy treatment and TOAST classification. Odds ratios for sHT were adjusted for atrial fibrillation, NIHSS and Thrombolysis/Thrombectomy treatment. HT, hemorrhagic transformation; PH, parenchymal hematoma; sHT, symptomatic hemorrhagic transformation; NIHSS, National Institutes of Health Stroke Scale score; TOAST, the Trial of ORG 10172 in Acute Stroke Treatment.

In our derivation cohort, 150 (12.4%) patients had all three biomarkers achieving the thresholds, and had the highest risk of HT. The patients with greater number of biomarkers achieving the thresholds had a higher risk of HT (*P* for trend <0.001, Figure 2). After adjustment for the confounders, compared to the patients without any biomarker achieving the thresholds, the ORs (95% CIs) of those with three biomarkers were 6.65 (3.06–14.48) for HT. Additionally, we assessed the effect of multiple biomarkers by constructing a continuous multi-biomarker score based on the estimates of beta coefficients of these three biomarkers obtained from logistic regression model (Figure 2). Patients with a higher score had an increased risk of HT (*P* for trend <0.001), and the adjusted OR (95% CI) for the highest tertile of the multi-biomarker score was 2.76 (1.75–4.35) for HT compared to patients with lowest tertile score. Similarly, the multi-biomarker score was also significantly associated with an increased risk of HT (adjusted OR, 2.32; 95% CI, 1.68–3.20, *P* < 0.001), independently of established covariates. Using a logistic regression model with restricted cubic splines, we found that a higher multi-biomarker score was associated with an increased risk of HT (Figure 3A).

Incorporating the three biomarkers (platelets, NLR, and HDL) simultaneously provided good discriminative capacity to predict HT (c statistic = 0.64; Table 4; likelihood ratio test *P* < 0.001) in the derivation cohort. Similarly, we also determined good discrimination of the three biomarkers in combination in the external validation cohort (c statistic = 0·70, 95% CI 0·58–0·82; Table 3). In both the derivation and validation cohorts, the three biomarkers in combination performed better in predicting HT than a single biomarker.

**Table 3:** Area under the receiver-operating characteristic curve (AUC) of biomarkers for predicting hemorrhagic transformation in derivation and validation cohorts.

	Derivation cohort (*n* = 1,207)		Validation cohort (*n* = 288)	
	AUC (95% CI)	*P*-value	AUC (95% CI)	*P*-value
Platelets (× 10^9^/L)	0.60 (0.56–0.65)	<0.001	0.59 (0.47–0.72)	0.157
Neutrophil-to-lymphocyte ratio	0.62 (0.58–0.67)	<0.001	0.73 (0.62–0.83)	<0.001<
High-density lipoprotein cholesterol (mmol/L)	0.57 (0.52–0.62)	<0.003	0.61 (0.49–0.74)	0.081
All three biomarkers	0.64 (0.60–0.69)	<0.001	0.70 (0.58–0.82)	<0.001<

**Table 4:** Reclassification of hemorrhagic transformation by circulating biomarkers among ischemic stroke patients.

Models	C statistics		Category-free NRI		IDI	
	Estimate (95% CI), %	*P*-value	Estimate (95% CI), %	*P*-value	Estimate (95% CI), %	*P*-value
Conventional model	0.78 (0.75–0.81)	Reference	Reference	Reference	Reference	Reference
Conventional model + PLT	0.79 (0.76–0.82)	0.115	49.3% (33.4%–65.2%)	<0.001	2.1% (0.8%–3.3%)	0.002
Conventional model + NLR	0.79 (0.75–0.82)	0.062	34.5% (18.6%–50.4%)	<0.001	1.0% (−0.2%–2.1%)	0.102
Conventional model + HDL	0.78 (0.75–0.82)	0.573	31.7% (15.8%–47.6%)	<0.001	1.4% (0.3%–2.5%)	0.013
Conventional model + multi-marker score	0.80 (0.77–0.83)	0.010	56.2% (40.4%–72.1%)	<0.001	3.2% (1.4%–5.0%)	0.001
Conventional model + all three biomarkers	0.80 (0.77–0.83)	0.012	65.6% (49.7%–81.4%)	<0.001	3.0% (1.5%–4.6%)	<0.001<

*Abbreviations: CI; confidence interval; NRI, net reclassification improvement; IDI, integrated discrimination index; PLT, Platelet; NLR, Neutrophil-to-lymphocyte Ratio; Conventional model included age, sex, atrial fibrillation, National Institutes of Health Stroke Scale score, smoking, drinking, systolic blood pressure, antiplatelets, lipid-lowering agents, endovascular treatment (Thrombolysis/Thrombectomy) and the Trial of ORG 10172 in Acute Stroke Treatment*.

### Multi-biomarkers and Association With Parenchymal Hematoma (PH) and Symptomatic Hemorrhagic Transformation (sHT)

We also assessed the effect of the multi-biomarker model for predicting the risks of PH and sHT, respectively in our derivation cohort. Patients with all three biomarkers achieving the thresholds had the highest risks of PH and sHT (all *P* for trend ≤0.001, Figure 2). Patients with a higher multi-biomarker score had an increased risk of PH (*P* for trend <0.001), and the adjusted OR (95% CI) for the highest tertile of multi-biomarker score was 2.68 (1.49–4.84) for PH compared to patients with lowest tertile of score (Figure 2). Similarly, an increased trend in the risk of sHT from the lowest tertile of score to the highest tertile of score was also found (*P* for trend = 0.001, Figure 2). In multivariate analysis, the multi-biomarker score was still significantly associated with sHT (adjusted OR, 2.28; 95% CI, 1.50–3.44; *P* = 0.008) and PH (adjusted OR, 2.39; 95% CI, 1.49–3.83; *P* < 0.001). Using a logistic regression model with restricted cubic splines, we found that a higher multi-biomarker score was associated with an increased risk of PH (Figure 3B) and sHT (Figure 3C).

### The Additional Predictive Value of Biomarkers Over the Conventional Model

A conventional model included age, sex, atrial fibrillation, National Institutes of Health Stroke Scale score, smoking, drinking, SBP, antiplatelets, lipid-lowering agents, thrombolysis/thrombectomy treatment and Toast classification was developed according to the multivariate logistic regression analysis for the association with HT in the derivation cohort. The predictive performance of the conventional model was significantly improved by adding three biomarkers simultaneously or the multi-biomarker score (both c statistic = 0.8, *P* = 0.01, Table 4). Simultaneously adding these three biomarkers offered the greatest positive reclassification for HT over the conventional model (category-free NRI 65.6%, *P* < 0.001, Table 4). Addition of the multi-biomarker score also confirmed an increased sensitivity (IDI, 3.2%; *P* = 0.001) and positive reclassification (NRI, 56.2%; *P* < 0.001; Table 4). Good performance of predicting PH and sHT was also found when three biomarkers or the multi-biomarker score were simultaneously added to the conventional model (Supplementary Table 3).

## Discussion

In the study, 17 circulating biomarkers representing different pathologic pathways after stroke were analyzed, and their predictive values for HT were evaluated. Individually, we found platelets, NLR, and HDL were related to the risk of HT, independently of established covariates. When we combined these three biomarkers together, there was a clear ascending trend in risk of HT, PH and sHT with increasing numbers of biomarkers. Addition of these three biomarkers simultaneously/the multi-biomarker score to the conventional risk model enhanced the risk refinement and reclassification for HT, PH and sHT. In addition, the multi-biomarker model was successfully externally validated and showed good discrimination for HT.

Prior studies (Bruno et al., 2002; Lee et al., 2010) demonstrated that individual biomarkers are associated with a gradient of risk for HT after adjusting for conventional risk factors. Our study confirmed prior results and investigated the relationship between various biomarkers and HT after AIS systematically. In this study, platelets, NLR, and HDL were identified to associate with HT independently. Despite that, the individual predictive value for predicting HT of the three identified biomarkers was modest. Given the multiple and interconnected pathological processes underlying HT (Álvarez-Sabín et al., 2013), one biomarker may not be able to capture sufficient information to assess the risk of HT. Recent studies (Melander et al., 2009; Jackson et al., 2016; Hijazi et al., 2017; Zhong et al., 2019) demonstrated the incremental usefulness of multiple biomarkers as a predictor for outcomes in patients with several cardiovascular conditions, and the risk of cardiovascular events. However, there are limited data on the predictive value of multiple biomarkers for the risk of HT after ischemic stroke.

A prospective cohort study (Marsh et al., 2016) investigated the addition of serum glucose, WBC count and warfarin use on admission to the HeRs score (age, infarct volume, eGFR) improved the predictive value for HT in 241 patients with AIS and with an indication for anticoagulation. We extended this finding and identified the addition of the three biomarkers (platelets, NLR, and HDL) that enhanced the risk prediction for HT compared to conventional risk model in a real-world practice. In addition, these three biomarkers we chose in our study represented different pathologic pathways underlying HT, which is consistent with the opinion (Wang, 2011; Bachmann and Wang, 2018) that multiple biomarkers from one pathway may provide overlapping information, resulting non-improvement in risk classification after addition of multi-biomarkers.

This study has several strengths. The sample size of the derivation cohort was larger than most of the previous studies of blood biomarkers and HT after ischemic stroke. Comprehensive information and multiple biomarkers were collected and measured with standardized protocols and rigid quality control procedure. Good performance of the multi-biomarker model in the external validation cohort and inclusion of a broad spectrum of people with ischemic stroke support the use of this instrument in diverse clinical settings. Furthermore, our study may have potential important implications for clinical practice. The three biomarkers are easily measured, and routinely available in the emergency department, which could shorten the turnaround time to their availability. In addition, our study suggests that addition of multi-biomarker in conjunction with established risk factors could refine risk prediction for HT. A biomarker panel predicting HT may affect clinical decisions regarding reperfusion and anticoagulation therapy. Although the multi-biomarker model had a good ability to identify patients at high risk of HT in the study, withholding reperfusion and anticoagulation therapy in those patients resulting in better long-term outcomes remains unknown. Further longitudinal studies with large sample size evaluating whether incorporating blood biomarkers into clinical decision could allow us to choose ischemic stroke patients who will benefit the most from reperfusion and anticoagulation therapy and avoid exposing other patients with substantial HT risk could bring interesting information and potentially help us get closer to the aim of precision medicine.

Several limitations of this analysis should be acknowledged. First, we did not include radiological markers such as lesion size and the Alberta Stroke Program Early CT (ASPECT) score, because it is not routinely determined in the clinical setting, and there is significant interobserver variability. Compared to anterior stroke, posterior stroke had a lower frequency of HT. Considering this, there might be some differences in the development of HT in ischemic stroke patients with different locations. The location of the infarcts has not been analyzed in the study. Future studies are needed to investigate whether the model have the similar performance in both anterior and posterior stroke. In addition, there are numerous other biomarkers that were not analyzed in the study. Despite that, the selected biomarkers were repeatedly used in previous studies and can be easily ascertained in different settings. They are part of the routine tests of stroke patients, making our results applicable for clinical practice. Second, we only included ischemic stroke patients with a follow-up CT/MRI within 7 days after admission to identify HT. Therefore, we only looked at HT within about 1 week after stroke onset in the study. Although, this may not reflect clinical practice in some settings, we aimed to detect more HT in the study. Our previous study (Tan et al., 2014) showed HT usually occurs within 1 week after stroke onset. Third, HT in the study was detected by a follow-up CT or MRI, and MRI is more sensitive than CT for detection of HT (Álvarez-Sabín et al., 2013). However, CT and MRI have a similar sensitivity to detect PH after ischemic stroke. In the study, the addition of the multi-biomarker score to a conventional model improved not only the risk refinement for HT, but also for PH. Fourth, there were differences in baseline characteristics and observed frequency of HT in the derivation and validation cohorts. This variance could be attributed to differences in hospital settings (central vs. local hospital). The multi-biomarker model performed well in different hospitals, showing the robustness and reliability of the model when applied to different cohorts. Finally, the biomarkers were measured only once, while serial measurements of these biomarkers may offer more information and help us to better understand the mechanism of HT after ischemic stroke. Future studies should unravel whether the association between these blood biomarkers and HT is only an epiphenomenon or whether it reflects a causal relationship.

## Conclusions

The present study suggests that levels of platelets, NLR, and HDL on admission were individually associated with an increased risk of HT after ischemic stroke, independent of established variables. Incorporation of a combination of these three biomarkers to a basic model with conventional risk factors enhanced the risk discrimination and reclassification for HT in patients with ischemic stroke. Our multi-biomarker model might be a useful instrument to identify individuals who are most likely to benefit from reperfusion and anticoagulation interventions.

## Data Availability Statement

The data that support the results of this study are available from the corresponding author upon reasonable request.

## Ethics Statement

The studies involving human participants were reviewed and approved by the Biomedical Research Ethics Committee of West China Hospital, Sichuan University [2016(339)]. The patients/participants provided their written informed consent to participate in this study.

## Author Contributions

JLiu drafted the manuscript. YW analyzed the data. YJ, WG, QS, CW, JLi, and SZ collected the data. ML designed the research. All authors contributed to the article and approved the submitted version.

## Conflict of Interest

The authors declare that the research was conducted in the absence of any commercial or financial relationships that could be construed as a potential conflict of interest.
