# Supplementary material for: Prediction of Hemorrhagic Transformation After Ischemic Stroke: Development and Validation Study of a Novel Multi-biomarker Model
Source: Front Aging Neurosci. 2021 May 28;13:667934. doi: 10.3389/fnagi.2021.667934 (PMC8193036; doi:10.3389/fnagi.2021.667934)
Supplement: Supplementary file 1 [file Table_1.DOCX]

**Supplemental Table 1 Baseline characteristics of study participants of** **derivation and validation cohorts**

|  | **Derivation cohort** | **Validation cohort** | **P values** |
| --- | --- | --- | --- |
|  | **n=1207** | **n=288** |  |
| **Demographics** |  |  |  |
| Age (years), mean (SD) | 67.22 (13.94) | 69.01 (11.98) | 0.045 ^c^ |
| Male, n (%) | 727 (60.23%) | 181 (62.85%) | 0.414 ^b^ |
| **Medical history** |  |  |  |
| Hypertension, n (%) | 656 (54.35%) | 169 (58.68%) | 0.184 ^b^ |
| Diabetes mellitus, n (%) | 246 (20.38%) | 53 (18.40%) | 0.451 ^b^ |
| Hyperlipidemia, n (%) | 38 (3.15%) | 21 (7.29%) | 0.001 ^b^ |
| Atrial fibrillation, n (%) | 207 (17.15%) | 21 (7.29%) | <0.001 ^b^ |
| **Therapy before admission** |  |  |  |
| Antiplatelets, n (%) | 138 (11.43%) | 36 (12.50%) | 0.612 ^b^ |
| Lipid-lowering agents, n (%) | 81 (6.71%) | 23 (7.99%) | 0.445 ^b^ |
| Anticoagulants, n (%) | 71 (5.88%) | 13 (4.51%) | 0.365 ^b^ |
| **Clinical features** |  |  |  |
| Onset to addition time (hours), median (IQR) | 5.00 (3.00-24.00) | 7.00 (3.00-17.00) | 0.137 ^a^ |
| Current smoking, n (%) | 485 (40.18%) | 94 (32.64%) | 0.018 ^b^ |
| Current drinking, n (%) | 286 (23.70%) | 76 (26.39%) | 0.338 ^b^ |
| NIHSS on admission, median (IQR) | 6.00 (3.00-14.00) | 4.00 (2.00-9.00) | <0.001 ^a^ |
| SBP (mmHg), mean (SD) | 147.61 (23.89) | 145.50 (132.00-164.00) | 0.265 ^c^ |
| DBP (mmHg), mean (SD) | 85.04 (15.87) | 81.00 (75.00-90.00) | 0.229 ^c^ |
| **Biomarkers** |  |  |  |
| Glucose (mmol/L), mean (SD) | 7.11 (6.03-8.81) | 6.69 (5.71-8.81) | 0.006 ^c^ |
| WBC count (× 10^9^/L), median (IQR) | 8.02 (3.24) | 7.99 (3.89) | <0.001 ^a^ |
| Monocyte count (× 10^9^/L), median (IQR) | 0.36 (0.27-0.47) | 0.44 (0.33-0.56) | <0.001 ^a^ |
| Neutrophils (× 10^9^/L), median (IQR) | 5.49 (4.10-7.62) | 4.93 (3.83-6.49) | <0.001 ^a^ |
| Lymphocytes, median (IQR) | 1.30 (0.95-1.80) | 1.24 (0.87-1.67) | 0.051 ^a^ |
| Neutrophil-to-lymphocyte Ratio, median (IQR) | 4.18 (2.58-7.21) | 4.08 (2.50-6.56) | 0.434 ^a^ |
| TG (mmol/L), median (IQR) | 1.28 (0.90-1.87) | 1.24 (0.88-1.78) | 0.418 ^a^ |
| TC (mmol/L), median (IQR) | 4.30 (3.63-5.01) | 4.39 (3.75-5.09) | 0.093 ^a^ |
| HDL (mmol/L), median (IQR) | 1.24 (1.00-1.49) | 1.30 (1.09-1.54) | 0.004 ^a^ |
| LDL (mmol/L), median (IQR) | 2.54 (1.97-3.19) | 2.60 (1.99-3.21) | 0.528 ^a^ |
| Platelets (× 10^9^/L), median (IQR) | 168.00 (132.00-207.00) | 162.50 (126.75-214.50) | 0.931 ^a^ |
| Bilirubin (10^-6 mol/l), median (IQR) | 11.30 (8.50-15.20) | 13.00 (10.00-17.33) | <0.001 ^a^ |
| Alanine aminotransferase (IU/L), median (IQR) | 19.00 (13.00-27.00) | 18.00 (14.00-25.00) | 0.224 ^a^ |
| Aspartate aminotransferase (IU/L), median (IQR) | 22.00 (18.00-27.00) | 22.00 (18.00-27.25) | 0.487 ^a^ |
| Alkaline phosphatase (IU/L), median (IQR) | 78.00 (65.00-94.00) | 66.00 (56.00-82.00) | <0.001 ^a^ |
| Serum creatinine, median (IQR) | 74.00 (62.00-88.50) | 68.45 (56.53-82.82) | <0.001 ^a^ |
| Cystatin C (mg/L), median (IQR) | 0.92 (0.80-1.13) | 0.96 (0.79-1.21) | 0.264 ^a^ |
| **Therapy after admission** |  |  |  |
| Antiplatelets, n (%) | 1087 (90.06%) | 275 (95.49%) | 0.004 ^b^ |
| Anticoagulants, n (%) | 177 (14.66%) | 35 (12.15%) | 0.272 ^b^ |
| Lipid-lowering agents, n (%) | 1094 (90.64%) | 273 (94.79%) | 0.024 ^b^ |
| Thrombolysis, n (%) | 130 (10.77%) | 57 (19.79%) | <0.001 ^b^ |
| Thrombectomy, n (%) | 121 (10.02%) | 14 (4.86%) | 0.006 ^b^ |
| EVT, n (%) | 221 (18.31%) | 68 (23.61%) | 0.041 ^b^ |
| **TOAST classification** |  |  | <0.001 ^b^ |
| Large-artery atherosclerosis, n (%) | 354 (29.33%) | 151 (52.43%) |  |
| Small-artery occlusion, n (%) | 260 (21.54%) | 68 (23.61%) |  |
| Cardioembolic, n (%) | 349 (28.91%) | 37 (12.85%) |  |
| Undetermined etiology, n (%) | 31 (2.57%) | 9 (3.12%) |  |
| Other etiology, n (%) | 213 (17.65%) | 23 (7.99%) |  |

Abbreviations: HT, hemorrhagic transformation; NIHSS, National Institutes of Health Stroke Scale; SBP, systolic blood pressure; DBP, diastolic blood pressure; WBC, white blood cell; TG, triglyceride; TC, total cholesterol; HDL, high-density lipoprotein cholesterol; LDL, low-density lipoprotein cholesterol; EVT, endovascular treatment (Thrombolysis/Thrombectomy); TOAST, the Trial of ORG 10172 in Acute Stroke Treatment.

a Mann-Whitney Test

b χ2 test

c Student’s test

**Supplemental Table 2 Sensitivity and specificity of biomarkers and risk of hemorrhagic transformation after ischemic stroke**

| **Biomarkers** | **AUC** | **Specificity** | **Sensitivity** | **OR (95% CI)** | **P value** |
| --- | --- | --- | --- | --- | --- |
| **Platelets (× 10^9^/L), ≤169** | 0.60 | 0.51 | 0.66 | 1.97 (1.37, 2.83) | <0.001 |
| **HDL (mmol/L), ≥ 1.28** | 0.57 | 0.56 | 0.58 | 1.64 (1.15, 2.33) | 0.006 |
| **Neutrophil-to-lymphocyte Ratio, ≥ 4.7** | 0.62 | 0.60 | 0.63 | 2.10 (1.47, 3.01) | <0.001 |
| **Neutrophils (× 10^9^/L), ≥ 5.83** | 0.59 | 0.57 | 0.60 | 1.68 (1.17, 2.40) | 0.005 |
| **Lymphocytes, ≤1.25** | 0.60 | 0.61 | 0.57 | 1.92 (1.35, 2.74) | <0.001 |

Abbreviations: Area under the receiver-operating characteristic curve (AUC), OR, odds radio; CI, confidence level; HDL, high-density lipoprotein cholesterol;

**Supplemental Table 3 Reclassification of parenchymal hematoma and symptomatic hemorrhagic transformation by circulating biomarkers among ischemic stroke patients**

|  | **Parenchymal hematoma** | | | | | |
| --- | --- | --- | --- | --- | --- | --- |
| **Models** | **C statistics** | | **Category-free NRI** | | **IDI** | |
|  | **Estimate (95% CI), %** | **P value** | **Estimate (95% CI), %** | **P value** | **Estimate (95% CI), %** | **P value** |
| **Conventional model** | 0.81 (0.78 - 0.85) | Reference | Reference | Reference | Reference | Reference |
| **Conventional model + PLT** | 0.82 (0.79 - 0.86) | 0.110 | 53.5% (33.4% - 73.7%) | <0.001 | 0.4% (-0.6% - 1.4%) | 0.442 |
| **Conventional model + NLR** | 0.82 (0.78 - 0.86) | 0.152 | 32.2% (12.0% - 52.4%) | 0.002 | 1.9% (-0.5% - 4.2%) | 0.113 |
| **Conventional model + HDL** | 0.81 (0.78 - 0.85) | 0.954 | 33.6% (13.4% - 53.8%) | 0.001 | 0.9% (-0.2% - 2.1%) | 0.116 |
| **Conventional model + multi-biomarker score** | 0.83 (0.80 - 0.86) | 0.009 | 48.8% (28.6% - 69.0%) | <0.001 | 1.0% (-0.4% - 2.4%) | 0.161 |
| **Conventional model + all 3 biomarkers** | 0.82 (0.79 - 0.86) | 0.155 | 64.7% (44.5% - 84.8%) | <0.001 | 1.7% (-0.1% - 3.4%) | 0.062 |
|  | **Symptomatic hemorrhagic transformation** | | | | | |
| **Models** | **C statistics** | | **Category-free NRI** | | **IDI** | |
|  | **Estimate (95% CI), %** | **P value** | **Estimate (95% CI), %** | **P value** | **Estimate (95% CI), %** | **P value** |
| **Conventional model** | 0.76 (0.70 - 0.83) | Reference | Reference | Reference | Reference | Reference |
| **Conventional model + PLT** | 0.79 (0.72 - 0.85) | 0.298 | 56.5% (26.4% - 86.6%) | <0.001 | 0.3% (-0.5% - 1.2%) | 0.457 |
| **Conventional model + NLR** | 0.78 (0.72 - 0.84) | 0.057 | 35.0% (4.9% - 65.1%) | 0.023 | 2.2% (-2.1% - 6.4%) | 0.313 |
| **Conventional model + HDL** | 0.77 (0.71 - 0.83) | 0.535 | 30.6% (0.4% - 60.7%) | 0.047 | 0.6% (-0.3% - 1.4%) | 0.186 |
| **Conventional model + multi-marker score** | 0.80 (0.74 - 0.86) | 0.060 | 67.3% (37.2% - 97.4%) | <0.001 | 2.4% (-1.9% - 6.7%) | 0.273 |
| **Conventional model + all 3 biomarkers** | 0.79 (0.73 - 0.84) | 0.188 | 71.3% (41.2% - 101.4%) | <0.001 | 1.7% (0.0% - 3.4%) | 0.049 |

Abbreviations: CI; confidence interval; NRI, net reclassification improvement; IDI, integrated discrimination index; PLT, Platelet; NLR, Neutrophil-to-lymphocyte Ratio; HDL, high-density lipoprotein cholesterol;

Conventional model for parenchymal hematoma age, sex, atrial fibrillation, National Institutes of Health Stroke Scale score, smoking, drinking, systolic blood pressure, antiplatelets, lipid-lowering agents, endovascular treatment (Thrombolysis/Thrombectomy) and the Trial of ORG 10172 in Acute Stroke Treatment.

Conventional model for symptomatic hemorrhagic transformation included atrial fibrillation, National Institutes of Health Stroke Scale score and endovascular treatment (Thrombolysis/Thrombectomy).
